# The Trauma Training Score: A Novel and Objective Method for Performance Evaluation

**DOI:** 10.7759/cureus.74102

**Published:** 2024-11-20

**Authors:** Scott E Young, Gerrit W Davis, Drew M Thomas, Tam N Pham, Kyle Couperus

**Affiliations:** 1 Department of Emergency Medicine, University of Washington School of Medicine, Seattle, USA; 2 Department of Emergency Medicine, Madigan Army Medical Center, Tacoma, USA; 3 Department of Statistics, The Geneva Foundation, Tacoma, USA; 4 Department of Surgery, University of Washington School of Medicine, Seattle, USA

**Keywords:** emergency simulation, trauma evaluation, trauma simulation, trauma team assessment, trauma training

## Abstract

Background: Assessing proficiency in the initial management of a traumatically injured patient is challenging. Previously developed scoring tools frequently looked to evaluate single domains of a trauma leader or team's performance. An updated multi-domain scoring tool is needed to evaluate individual and group performance in the initial phases of trauma resuscitation.

Methods: The modified Delphi technique was used to improve and obtain consensus on a multi-domain trauma training score (TTS). Subject matter experts (SMEs) in trauma across the United States were contacted electronically to consider voluntary participation in this study. Elective participants electronically received and commented on statements related to the 10 separate domains of a proposed TTS. These statements were evaluated using a 5-point Likert scale ranging from "Strongly Agree" to "Strongly Disagree". Feedback from the SMEs was used to modify each domain that did not receive consensus, and repeat iterations were performed until 80% or greater consensus was achieved. Internal consistency was measured using Cronbach’s-ɑ, with a goal of greater than 0.8.

Results: Twenty-eight SMEs elected to participate in the modified Delphi process representing emergency medicine, trauma surgery, and critical care. Consensus was achieved when 80% or greater of the SMEs responded with a 4 or a 5 to each statement. Four total rounds of review and modification were required to achieve consensus on all statements. Cronbach’s-ɑ for each round was greater than 0.85.

Discussion: A unifying standardized outcome for measuring performance in the initial phases of trauma resuscitation is needed. The TTS developed in this study used expert consensus to provide a multi-domain means of evaluating trauma practitioners of all levels in both live and simulated patients.

## Introduction

Traumatic injuries account for a significant burden of global morbidity and mortality. The World Health Organization (WHO) estimates that, in 2019, over 4.4 million people died from unintentional and violence-related injuries. This accounted for 8% of all deaths and is the primary cause in approximately 10% of all years lived with a disability worldwide [1]. Preparing trauma team leaders at all levels of training to meet the challenges of trauma resuscitation requires validated teaching and assessment techniques [2].

The Advanced Trauma Life Support (ATLS), Tactical Combat Casualty Care (TCCC), Rural Trauma Team Development Course (RTTDC), and Disaster Management and Emergency Preparedness (DMAP) courses all provide excellent initial training in trauma and disaster management [3-5]. Continued development of these skills can be accomplished via simulation-based training, with published improvements in metrics such as time to intervention, provider knowledge, and provider satisfaction [6,7].

One of the current gaps in trauma resuscitation assessment is the lack of a comprehensive scoring tool that evaluates multiple domains of an individual or group's performance during the initial phases of care. Separate tools in the domains of team leader performance, team communication, and time-sensitive critical actions have previously been developed but have yet to be combined and validated by anonymized expert consensus [8,9]. Additionally, domains covering the assessment of an up-to-date approach to trauma-centric areas such as massive external hemorrhage, airway management, hypothermia prevention, and head injury are not available in the current literature.

Holcomb et al. published the validation of an excellent trauma team evaluation tool in 2002 [10]. The assessment and management of traumatic injuries during the initial phases of care have progressed significantly in the last 20 years. Additionally, the technology utilized to train and assess trauma providers has changed dramatically, including augmented and virtual reality [11]. An updated scoring tool is needed to account for the evolution of both trauma patient care and methods of training trauma providers. The objective of this study is to use expert consensus to develop a multi-domain trauma training score (TTS) that can be used in both simulation and live patient scenarios to evaluate all levels of trauma practitioners.

## Materials and methods

A modified Delphi technique was used to collect feedback and achieve expert consensus on all aspects of the TTS. The modified Delphi technique is a process that uses a series of anonymous questionnaires to gather and reach an agreement on subject matter expert (SME) opinion [12]. Each round of the questionnaire changes based on the feedback of SMEs until consensus is achieved. The modified Delphi technique is a common technique used to develop best practice guidelines and is an ideal way to create an expert-derived scoring tool to assist in the evaluation of trauma practitioners [13].

Approval for this study was obtained from our Institutional Review Board. Study investigators identified SMEs through both military and civilian institutions nationwide. The participants were selected based on their well-known expertise in the field of trauma management based on academic and clinical accomplishments. Each potential SME was contacted electronically to request their voluntary participation in this process. Responses were collected, and a database was created with all consenting SMEs. Participants were required to be board-certified in surgery, critical care medicine, or emergency medicine, with at least one year of post-residency or fellowship experience. 

The participating SMEs then received an email containing the most up-to-date version of the TTS and a link to an anonymous REDCap© survey. The initial items of the survey were demographic questions regarding the respondents' specialty, years of post-residency practice, and military experience if relevant. The next part of the survey consisted of each statement to be evaluated for consensus, along with a 5-point Likert scale ranging from "Strongly Agree" to "Strongly Disagree" for each statement. For example, the SME would see the statement "The listed categories capture the appropriate initial trauma resuscitation domains for an evaluation tool that can be used to score simulation participants", followed by the described Likert scale. A survey response of 3 (neutral) or less initiated a drop-down box for SME feedback. The survey then continued until the SME reviewed all remaining statements requiring consensus. A comments box was placed at the end of the survey to gather additional suggestions.

A consensus was achieved on survey items when ≥80% of responses to the statement were 4 or 5, corresponding with "Agree" and "Strongly Agree". Eighty percent consensus was chosen based on a review of the literature discussing the general approach to the modified Delphi process, as well as previous examples of its use in clinical assessment tools [14-16]. Items were removed from the REDCap© survey once they met consensus, and SME feedback was used to modify the TTS. These modifications might include adding, removing, or changing items from a particular domain of the TTS. Iterations of anonymous electronic surveys, assessment of feedback, and modification of the TTS were repeated until ≥80% consensus had been achieved for all domains and items. Cronbach’s-ɑ was calculated at the end of each iteration and was used to measure adequate internal consistency among survey respondents, with a goal of ≥ 0.8.

## Results

Twenty-eight SMEs agreed to participate in the modified Delphi process, consisting of 14 emergency medicine physicians (50%), 11 surgeons who care for trauma patients (39%), and three non-surgical trauma critical care physicians (11%). A total of four rounds of review and modification were required to meet consensus for all elements of the TTS.

Twenty-one of the 28 SMEs (75%) participated in round 1 of the Delphi process. See Table 1 for the distribution of responding SMEs for each round of the Delphi process. The average years of post-residency experience for the SMEs participating in each round can be found in Table 2.

**Table 1:** Distribution of the responding subject matter experts for each round of the modified Delphi process Table Credits: Scott Young, Gerrit Davis, and Kyle Couperus

Modified Delphi Iteration	Specialty	N (%)
Round 1	Emergency Medicine	12/21 (57.1%)
	General Surgery	4/21 (19.0%)
	Critical Care	4/21 (19.0%)
	Cardiothoracic Surgery	1/21 (4.8%)
Round 2	Emergency Medicine	11/20 (55.0%)
	General Surgery	7/20 (35.0%)
	Critical Care	1/20 (5.0%)
	Cardiothoracic Surgery	1/20 (5.0%)
Round 3	Emergency Medicine	8/14 (57.1%)
	General Surgery	3/14 (21.4%)
	Critical Care	1/14 (7.1%)
	Cardiothoracic Surgery	1/14 (7.1%)
	Vascular Surgery	1/14 (7.1%)
Round 4	Emergency Medicine	9/15 (60.0%)
	General Surgery	4/15 (26.6%)
	Critical Care	1/15 (6.7%)
	Cardiothoracic Surgery	1/15 (6.7%)

**Table 2:** Average years of post-residency training experience of the responding subject matter experts for each round of the modified Delphi process Table Credits: Scott Young, Gerrit Davis, and Kyle Couperus

Modified Delphi Iteration	Number of Responding Subject Matter Experts	Mean Years of Post-Residency Experience
Round 1	21	9.1 (2 to 18 years)
Round 2	20	8.6 (2 to 19 years)
Round 3	14	8.5 (3 to 19 years)
Round 4	15	9.9 (3 to 20 years)

Cronbach’s-ɑ was 0.86 for round 1, indicating adequate internal consistency for the survey. Five statements from the TTS had greater than 80% consensus during round 1 with a rating of 4 or 5 from the SMEs: “The listed categories capture the appropriate initial trauma resuscitation domains for an evaluation tool that can be used to score simulation participants" (e.g., team leader performance, massive hemorrhage, airway, respiration, circulation, hypothermia, head/C-spine injury, disposition, and harmful actions) (18 of 21 participants, 85.7%). “The actions listed under the ‘Airway’ and ‘Respiration’ domains are appropriately divided between the two categories” (19 of 21 participants, 90.5%). The "Airway" and "Respiration" domains are shown in Tables 3-4, respectively.

**Table 3:** Trauma training score: "Airway" domain Table Credits: Scott Young, Gerrit Davis, and Kyle Couperus

Airway	Score: ____out of____possible points (Max of 8)
1. Assesses airway	0 = No assessment 2 = Misses key portion of assessment 4 = Talks to the patient, completely assesses airway to include any field interventions
2. Performs basic airway interventions (oxygen, airway adjuncts, prepares for further intervention, etc.)	0 = Indicated, does not perform 1 = Indicated, delayed or incorrect performance 2 = Correctly performed, appropriate timing
3. Performs advanced airway intervention(s)	0 = Indicated, does not perform 1 = Indicated, delayed or incorrect performance 2 = Correctly performed, appropriate timing
Comments:	

**Table 4:** Trauma training score: "Respiration" domain Table Credits: Scott Young, Gerrit Davis, and Kyle Couperus

Respiration	Score: ____out of____possible points (Max of 10)
1. Assesses respiration	0 = No assessment 2 = Breathing observed but no assessment for adequacy 4 = Full respiration assessment
2. Recognizes and treats tension pneumothorax	0 = Does not recognize 1 = Recognizes, but delayed or incorrect intervention 2 = Correctly performed intervention, appropriate timing
3. Recognizes and treats open pneumothorax	0 = Does not recognize 1 = Recognizes, but delayed or incorrect intervention 2 = Correctly performed intervention, appropriate timing
4. Applies oxygen therapy based upon respiratory distress or pulse oximetry, if appropriate to the scenario	0 = Not recognized, not performed 1 = Recognized, delayed performance or not performed 2 = Recognized and performed
Comments:	

“The five items under the current ‘Trauma Team Performance’ domain cover all the areas a trauma team should be evaluated on” (17 of 21 participants, 81.0%). “The items listed in the ‘Hypothermia’ domain adequately address all the important interventions related to hypothermia identification and management” (18 of 21 participants, 85.7%). “The items listed under the ‘Harmful Actions (If Occurred)’ domain provide an appropriate modification to the trainees' score if mistakes are made, or interventions are missed” (19 of 21 participants, 90.5%). Table 5 shows the "Harmful Actions" domain.

**Table 5:** Trauma training score: "Harmful Actions" domain Table Credits: Scott Young, Gerrit Davis, and Kyle Couperus

Harmful Actions (If Cccurred)	
1. Minor action	(-) 2 per action = adverse event leading to potential or actual minor injury
2. Major action	(-) 8 = adverse event leading to potential or actual loss of life, limb or eyesight

Several modifications were made to the “Trauma Team Performance” and “Hypothermia” domains based on SME feedback despite reaching consensus. These items were resubmitted for SME evaluation in round 2. Additionally, a separate domain of “Optional Assessment and Treatment Adjuncts” was created to group specific items from the tool based on SME feedback.

Twenty of the 28 (71.4%) SMEs completed the second round of the Delphi process with Cronbach’s-ɑ of 0.92. The statements that met consensus during round 2 were as follows: “The actions required in the evaluation and management of massive hemorrhage are adequately covered under the current 'Massive Hemorrhage' domain" (items 1-9) (16 of 20 participants, 80.0%). Table 6 demonstrates the "Massive Hemorrhage" domain.

**Table 6:** Trauma training score: "Massive Hemorrhage" domain Table Credits: Scott Young, Gerrit Davis, and Kyle Couperus

Massive Hemorrhage	Score: ____ out of ____ possible points (Max of 10)
1. Recognizes significant hemorrhage	0 = Does not recognize or address 2 = Recognized but inadequately addressed 4 = Recognized and addressed
2. Applies direct pressure to stop bleeding	0 = Indicated, not applied 1 = Indicated, applied, but not in right order or untimely 2 = Indicated, applied in appropriate order and timely
3. Applies tourniquet to stop bleeding	0 = Indicated, not applied 1 = Indicated, applied, but not in right order or untimely 2 = Indicated, applied in appropriate order and timely
4. Applies junctional tourniquet and deep wound packing as appropriate to the scenario	0 = Indicated, not applied 1 = Indicated, applied, but not in right order or untimely 2 = Indicated, applied in appropriate order and timely
Comments:	

“The items listed in the 'Hypothermia' domain adequately address all the important interventions related to hypothermia identification and management” (17 of 20 participants, 85.0%). Table 7 shows the "Hypothermia" domain.

**Table 7:** Trauma training score: "Hypothermia" domain Table Credits: Scott Young, Gerrit Davis, and Kyle Couperus

Hypothermia	Score: ____ out of ____ possible points (Max of 6)
1. Covers patient after exposure	0 = Indicated, not completed 1 = Indicated, delayed or incorrect performance 2 = Indicated, correctly performed, appropriate timing
2. Applies warming device to intravenous (IV) fluids/blood	0 = Indicated, not completed 1 = Indicated, delayed or incorrect performance 2 = Indicated, correctly performed, appropriate timing
3. Applies warming device to the patient (Hypothermia Prevention and Management Kit or other)	0 = Indicated, not completed 1 = Indicated, delayed or incorrect performance 2 = Indicated, correctly performed, appropriate timing
Comments:	

“The 'Disposition' domain adequately covers all aspects of patient disposition” (18 of 20 participants, 90.0%). Table 8 shows the "Disposition" domain.

**Table 8:** Trauma training score: "Disposition" domain Table Credits: Scott Young, Gerrit Davis, and Kyle Couperus

Disposition	Score: ____out of ____ possible points (Max of 4)
1. Reassesses patient and interventions	0 = Not performed 1 = Reassesses patient or interventions 2 = Reassesses patient and interventions
2. Correct disposition (operating room, transfer, discharge, admission, etc.)	0 = Incorrect disposition 1 = Better disposition available 2 = Selects best disposition
Comments:	

“The five items under the current 'Trauma Team Performance' domain covers all the areas a trauma team should be evaluated on” (18 of 20 participants, 90.0%). Table 9 shows the "Trauma Team Performance" domain.

**Table 9:** Trauma training score: "Trauma Team Performance" domain Table Credits: Scott Young, Gerrit Davis, and Kyle Couperus

Trauma Team Performance	Score: ____ out of ____ possible points (Max of 10)
1. Team leadership is effective	0 = No delegation, no instruction 1 = Intermittent delegation and instruction 2 = Assigns roles, delegates/instructs throughout
2. Team members assume functional roles, pre-brief team	0 = Multiple uninvolved personnel 1 = 1 or 2 uninvolved personnel 2 = All personnel participate actively
3. Verbal communication with the team	0 = No clear instructions 1 = Incompletely verbalizes primary survey and trauma team priorities 2 = Consistent closed loop communication; debriefs team after scenario communication
4. Systematic and orderly assessment	0 = Disorganized 1 = VerticaI resuscitation (step by step approach) 2 = Horizontal resuscitation (simultaneous cooperative work by multiple providers)
5. Ability to handle distractions and maintain a calm demeanor	0 = Ineffectively controls emotions, allows attention to be frequently diverted 1 = Emotions and/or distractions somewhat hinder performance 2 = Maintains calm demeanor, appropriately manages distractions
Comments:	

Valuable SME feedback led to changes in the “Massive Hemorrhage” domain even though it reached consensus. This domain was resubmitted in round 3 for evaluation.

Fourteen of the 28 (50%) SMEs completed the third round of the Delphi process with Cronbach’s-ɑ of 0.93. The statements that met consensus during round 3 were as follows: “The actions required in the evaluation and management of massive hemorrhage are adequately covered under the current 'Massive Hemorrhage' domain (items 1-9)” (13 of 14 participants, 92.9%). “The actions required in the evaluation and management of circulation are adequately covered under the current 'Circulation' domain (items 1-7)” (12 of 14 participants, 85.7%). The "Circulation" domain is shown in Table 10.

**Table 10:** Trauma training score: "Circulation" domain Table Credits: Scott Young, Gerrit Davis, and Kyle Couperus

Circulation	Score: ____ out of ____ possible points (Max of 16)
1. Assesses vital signs, peripheral/central pulses	0 = Indicated, not completed 2 = Indicated, delayed or incorrect performance 4 = Indicated, correctly performed, appropriate timing
2. Obtains appropriate vascular access	0 = Indicated, not completed 1 = Indicated, delayed or incorrect performance 2 = Indicated, correctly performed, appropriate timing
3. Performs complete exposure for injuries/bleeding (includes rolling patient to assess back)	0 = Does not perform 1 = Incomplete exposure 2 = Complete exposure
4. Applies pelvic binder	0 = Indicated, not applied 1 = Indicated, applied, but not in right order or untimely 2 = Indicated, applied in appropriate order and timely
5. Orders blood products	0 = Indicated but not performed 1 = Indicated and performed but incorrect ratio, product(s), or process 2 = Indicated and appropriate ratio, product, and process
6. Administers Tranexamic Acid (TXA)	0 = Indicated but not performed 1 = Inappropriate dosing, preparation, or administration 2 = Given, appropriate dosing, preparation, and administration
7. Recognition of femur fractures	0 = Fractu re not identified 1 = Identified but not addressed 2 = Identified and addressed
Comments:	

“Items 1-6 under the 'Head/Spine Injury' domain cover the most important aspects in the basic assessment and intervention of potential head and spine injury” (13 of 14 participants, 92.9%). “The 'Optional Assessment and Treatment Adjuncts' domain covers all miscellaneous actions that do not fit elsewhere within this tool” (13 of 14 participants, 92.9%). Tables 11-12 illustrate the "Head/Spine Injury" and "Optional Assessment and Treatment Adjuncts" domains, respectively.

**Table 11:** Trauma training score: "Head/Spine Injury" domain Table Credits: Scott Young, Gerrit Davis, and Kyle Couperus

Head/Spine Injury	Score: ____ out of ____ possible points (Max of 12)
1. Assesses level of consciousness	0 = Not completed 1 = Delayed or incorrect performance 2 = Correctly performed appropriate timing [GCS]
2. Pupil assessment	0 = Not performed 2 = Performed
3. Assesses for neurologic deficit	0 = Not performed 1 = Partial assessment 2 = Performed
4. Performs appropriate interventions as indicated for traumatic brain injury (i.e., elevate the head of the bed or reverse Trendelenburg, maintain appropriate blood pressure, avoid hypoxemia)	0 = Indicated, not completed 1 = Indicated, delayed or incorrect performance 2 = Indicated, correctly performed, appropriate timing
5. Maintains spinal precautions	0 = Indicated, not completed 1 = Indicated, delayed or incorrect performance 2 = Indicated, correctly performed appropriate timing
6. Hypertonic Saline and/or Mannitol administration	0 = Indicated, not completed 1 = Indicated, delayed or incorrect administration 2 = Indicated, correctly administered, appropriate timing
Comments:	

**Table 12:** Trauma training score: "Optional Assessment and Treatment Adjuncts" domain Table Credits: Scott Young, Gerrit Davis, and Kyle Couperus

Optional Assessment and Treatment Adjuncts	Score: ____ out of ____ possible points (Max of 20)
1. Extended Focused Assessment with Sonography for Trauma (EFAST) performance	0 = Indicated, not completed 1 = Indicated, delayed or incorrect performance 2 = Indicated, correctly performed, appropriate timing
2. Trauma Series X-Ray performance	0 = Indicated, not completed 1 = Indicated, delayed or incorrect performance 2 = Indicated, correctly performed, appropriate timing
3. Tetanus Vaccine administration	0 = Indicated, not completed 1 = Indicated, delayed or incorrect administration 2 = Indicated, correctly administered, appropriate timing
4. Antibiotics administration	0 = Indicated, not completed 1 = Indicated, delayed or incorrect administration 2 = Indicated, correctly administered, appropriate timing
5. Pain control and sedation administration	0 = Indicated, not completed 1 = Indicated, delayed or incorrect administration 2 = Indicated, correctly administered, appropriate timing
6. Extremity Splinting performance	0 = Indicated, not completed 1 = Indicated, delayed or incorrect performance 2 = Indicated, correctly performed, appropriate timing
7. Documentation of patient care	0 = Not performed 1 = Incomplete 2 = Com pleted
8. Obtains Relevant History	0 = Not performed 1 = Incomplete 2 = Completed
9. Resuscitative Endovascular Balloon Occlusion of the Aorta (REBOA), thoracotomy or other hemorrhage control procedure	0 = Indicated, not completed 1 = Indicated, delayed or incorrect performance 2 = Indicated, correctly performed, appropriate timing
10. Assesses for and treats ocular injury	0 = Indicated, not completed 1 = Indicated, delayed or incorrect performance 2 = Indicated, correctly performed, appropriate timing
Comments:	

Fifteen of the 28 (53.6%) SMEs completed round 4 of the Delphi process. The final statement that met consensus was the following: “There are no additional actions that I would recommend related to managing the airway and/or ventilation than what is listed in 'Airway' and 'Respiration'" (12 of 15 participants, 80.0%).

All of the domains and details of the TTS met consensus from the pool of SMEs after four iterations through the modified Delphi process. This included the addition of the "Optional Assessment and Treatment Adjuncts" domain and the details contained within it.

## Discussion

There is a need for a standardized outcome measure in trauma resuscitation performance. To our knowledge, this is the first expert consensus-created score designed to provide comprehensive feedback on multiple domains in the initial phases of trauma resuscitation. The combination of the domains listed in Tables 3-12 forms the three-page TTS, a performance assessment tool that has applications in both simulation and live patient care scenarios. Simulation settings may include live actor, mannequin, augmented/virtual/mixed-reality-based models, or a combination of those modalities. The TTS can be utilized in assessing a wide variety of medical practitioners including prehospital medical personnel, nurses, medical students, residents, fellows, and attending physicians of any specialty that manage trauma patients during the initial phases of care.

The initial phase of resuscitation in trauma includes managing the airway, stopping uncontrolled bleeding, addressing breathing and respiratory issues, assessing for neurologic disability, completely exposing the patient, and controlling the environment. Multiple mnemonics have been derived to facilitate recall and ensure consistent performance of these tasks, including “ABCDE” advocated by ATLS, and “MARCH” as described in the TCCC guidelines. The order of these tasks may sometimes be debated, but existing training programs agree that all these areas must be included in the initial evaluation of the trauma patient. The TTS is generally ordered according to the “MARCH” algorithm, but it is up to the end user to decide what the order of precedence is. The user of this trauma scoring tool could modify the order of the domains to whatever pattern best fits their training goals, as the SMEs validated the domains and the content of each domain. The SMEs were not asked to specify a particular order for the domains. Creating a fixed order would potentially limit the use of this training tool to practitioners and institutions that use a specific sequence of events in the initial phases of managing traumatic injuries. The goal of the TTS is to ensure all key elements of the initial phases of trauma resuscitation are evaluated while allowing the evaluator the freedom to determine the order of precedence of assessments and interventions as defined by the particular scenario.

Previous use of the Delphi Method includes the development of a technical skills evaluation tool in surgery by Palter et al. [14]. In this study, the authors derived an objective tool for the evaluation of laparoscopic right-sided and sigmoid colectomy with the feedback of 18 SMEs. Palter et al. then validated this tool's interrater reliability and validity in a later study by using it to assess the performance of novices versus experts [15]. This process most closely matches the goal of developing the TTS, and therefore a similar approach was used in this study. The initial statements submitted to the SMEs were created from existing ATLS and TCCC guidelines [3,4]. Each iteration of the process led to multiple modifications across each domain based on the feedback of the SMEs. The final consensus from the participating SMEs, representing both surgical and non-surgical trauma practitioners, ensured that all key elements of trauma resuscitation were included in the TTS.

The original trauma assessment tool created by Holcomb et al. used time-based metrics for each major intervention [10]. Feedback from our SMEs highlighted that the appropriate time for assessment and intervention could be affected by the specifics of the case. For instance, performing a needle thoracostomy may be "delayed" in one case due to the need to manage uncontrolled extremity hemorrhage, but it might be one of the first critical actions in another case limited to penetrating thoracic trauma. As a result of this feedback, we opted to give the evaluator more control over the scoring of timing based on their perspective of how a case should progress. This allows for maximum flexibility in scoring while ensuring that the key elements of trauma management are performed.

One of the limitations of this study is the loss of SMEs across the four iterations of the review. Trauma experts are busy, and it is difficult to ensure consistent participation, especially in the anonymized format utilized for this study. We believe that enough expert reviews were obtained to create a consensus-validated scoring tool despite the declining numbers of SMEs throughout the study process. Another limitation of our SME selection is the lack of international participation. Future studies utilizing SMEs outside of the United States could help further refine the TTS for a more worldwide application. Finally, evaluation by non-physician SMEs could help tailor the TTS to specific groups, including prehospital personnel.

Another challenge of the TTS is the need to have a grader with some experience in trauma management. As discussed above, the TTS provides a great deal of flexibility in scoring the order of events without compromising the key details of trauma resuscitation. As a result, the evaluator utilizing the TTS will require an understanding of how a trauma team should perform, the indications for procedures and when they should occur, and how harmful actions should affect a case. In augmented/virtual/mixed-reality-based models, these elements could be preprogrammed and thus obviate the need for an “expert.” In other simulation models, these specifics could be predetermined for each case so that a less experienced grader might be utilized.

## Conclusions

Our TTS, optimized through the modified Delphi process by a multidisciplinary group of trauma SMEs, provides a consensus-derived means of multi-domain assessment for trauma practitioners of all levels managing simulated or live patients. Future research should look to validate the TTS using scoring results in combination with learner and instructor feedback. Investigators should evaluate this data across various simulation models and learner levels with the final goal of improving the outcomes of trauma patients.
